# Homogeneity Parameter in Contrast-Enhanced Ultrasound Imaging Improves the Classification of Abnormal Cervical Lymph Node after Thyroidectomy in Patients with Papillary Thyroid Carcinoma

**DOI:** 10.1155/2019/9296010

**Published:** 2019-11-26

**Authors:** Jia Zhan, Xue-Hong Diao, Yue Chen, Wen-Ping Wang, Hong Ding

**Affiliations:** ^1^Department of Ultrasound, Zhongshan Hospital, Fudan University, Shanghai, China; ^2^Department of Ultrasound, Huadong Hospital, Fudan University, Shanghai, China

## Abstract

**Objective:**

To explore the conventional and contrast-enhanced ultrasound (CEUS) features of cervical lymph node metastasis (CLNM) in papillary thyroid carcinoma (PTC) patients postoperatively and analyze its pathological basis.

**Materials and Methods:**

Conventional and CEUS were performed in 86 abnormal cervical lymph nodes (ACLNs) from 56 PTC patients who had received thyroidectomy. Then, fine-needle aspiration (FNA) was taken to confirm pathological results, a multivariate analysis was performed to correlate the sonographic features of the CLNM, and then an equation for CLNM was established.

**Results:**

Fifty-four lymph nodes were confirmed to be metastasis of PTC by FNA. Intensity at peak time, homogeneity, and color flow patterns, cystic change, or microcalcification and echogenicity were significantly associated with CLNM. Multivariate analysis showed three strongest features (homogeneity, intensity of peak, and cystic change or calcification) to be significantly associated with the evidence of CLNM. Then, the equation was established with the following significant predictive factors: *P* = 1/1 + exp∑[−3.213 + 2.77 *∗* cystic or calcification + 0.13 *∗* CDFI patterns + 3.65 *∗* homogeneity + 2.43 *∗* intensity at peak time].

**Conclusion:**

Depiction of a heterogeneous hyperenhancement of cervical lymph nodes within CEUS studies and cystic change or microcalcification in conventional ultrasound were identified as predictive for metastatic lymph node invasion, and the equation was more accurate for predicting CLNM compared to single B-mode ultrasound and CEUS feature.

## 1. Introduction

Papillary thyroid carcinoma (PTC) is frequently associated with synchronous or cervical lymph node metastasis (CLNM), which caused regional recurrence [1]. CLNM commonly appears firstly in the central neck and subsequently in the lateral neck after thyroidectomy [2]. Conventional ultrasound (US) is recommended by the American Thyroid Association (ATA) guidelines for preoperative evaluation and postoperative follow-up of CLNM in PTC patients [3]. Present literature reveals the representative US features of CLNM, such as hyperechogenicity, presence of calcifications, loss of fatty hilum, cystic change, and round shape showing high sensitivity but low specificity in differentiating between benign and CLNM [4, 5]. In a word, there were limitations for conventional US to predict CLNM. Therefore, we need a new sonographic method to estimate CLNM in PTC patients after thyroidectomy.

Contrast-enhanced ultrasound (CEUS), which could study the diffuse pattern and alterations of nodal perfusion, has been widely used for differentiating between benign and malignant thyroid nodules [6, 7]. Recently several papers report that CEUS also shows a higher degree of diagnostic accuracy than conventional US on CLNM assessment preoperatively [8]. However, as far as we know, there were few CEUS studies evaluating the postoperative follow-up patients with cervical lymph nodes.

The aim of the present study was to estimate the performance of conventional US and CEUS in the diagnosis of CLNM and to explain the pathologic basis of CEUS performance.

## 2. Materials and Methods

### 2.1. Patients

Informed consent was obtained from all patients and the study was performed in accordance with the ethical guidelines of the Helsinki Declaration and approved by the local ethics committee. From March 2016 to November 2018, medical records of 218 consecutive TC patients after thyroidectomy were involved. Finally, fifty-six patients (16 men and 40 women) with 86 abnormal cervical lymph nodes (ACLNs) were enrolled in the study. The mean age of the examined patients was 52.6 ± 13.5 years (age range, 20–79 years). The median size of the ACLN was 1.2 × 0.7 × 1.0 cm (range, 0.5 × 0.4 × 0.4 cm to 2.5 × 1.5 × 1.8 cm).

The inclusion criteria for CEUS were as follows: (1) PTC patient after thyroidectomy and (2) cervical ACLN detected by conventional US in consecutive follow-up. ACLN was defined as nodes showing at least two following suspicious US features: (1) loss of fatty hilum, (2) presence of calcification or cystic change, (3) hyperechogenicity, and (4) round shape (length/width <2 : 1).

### 2.2. Conventional US (B-Mode and Doppler)

All conventional US scans were performed with a 9–12 MHz transducer (Acuson S2000, Siemens, Germany; Aplio 400, Toshiba, Japan). ACLNs were evaluated for size, distribution (level I to level VI), shape (oval or round), fatty hilum (present or loss) echogenicity (hypo- or hyperecho), cystic change (presence or absence), and microcalcification (presence or absence).

After grayscale US, color Doppler flow imaging (CDFI) was performed. The CDFI distribution of ACLN was classified into four types [9]: Type I: none; Type II: a hilar type (a central hilar vessel with or without centrifugal branches; Type III: an activated hilar type (a single vascular pole, wider and longer than the previous one, with some large branches); Type IV: peripheral type (the presence of peripheral vessels arranged into single poles with multiple irregular and tortuous centripetal branches).

### 2.3. CEUS Examination

All CEUS examination was performed by two senior clinical ultrasound examiners (J. Z. and X.-H. D.), with more than 8 years of experience in ultrasound thyroid examination. After the conventional US, the largest diameter of the nodule was chosen before the CEUS mode was switched. The focus zone was always placed at the bottom level of the nodule, and CEUS was performed using low mechanical index (MI < 0.10). Contrast agents (SonoVue, Bracco International, Milan, Italy) were injected intravenously as a bolus at a 1.2 mL dose, followed by a 5 mL saline flush. The timer was started during the CEUS process, and the images that lasted at least 2 min were digitally stored as raw data. Due to prior thyroidectomy, ACLNs on CEUS were evaluated individually, not relative to surrounding tissue. The entrance mode on CEUS was classified as centripetal, or noncentripetal; the echo intensity at peak enhancement was classified as hyper- or hypoenhancement; the homogeneity of enhancement was classified as homogeneous or heterogeneous.

### 2.4. Fine-Needle Aspiration (FNA)

US-guided FNA was performed simultaneously on ACLN with suspicious US features in our institution. FNA was performed with a 23-gauge needle attached to a 2 mL disposable plastic syringe and aspirator. Materials obtained from aspiration were smeared onto glass slides. All smears were immediately placed in 95% alcohol for Papanicolaou staining, and the remaining material was rinsed in saline for cell block processing. Cytopathologists were not on-site during the biopsy.

### 2.5. Statistical Analysis

Continuous quantitative data were expressed as mean ± standard deviation (SD). *χ*^2^ test was used to compare the categorical variables of clinical characteristics. A forward stepwise multivariate logistic regression analysis was performed to determine the independent factors associated with CLNM and all variables were adjusted. Odds ratios (ORs) with 95% confidence intervals (CIs) were calculated and binormal receiver operating characteristic (ROC) curves were analyzed for factors with significance on multivariate logistic regression analysis.

All statistical tests were performed using commercially available software (Stata version 10.0; Stata Corp, College Station, TX, USA). For all tests, a *P* value <0.05 was considered to indicate a statistically significant difference.

## 3. Results

### 3.1. Pathological Findings

There were 54 metastatic (62.8%) and 32 benign (37.2%) lymph nodes at final cytopathology. Fifty-four CLNMs originated from 33 patients; as a result, unilateral modified neck dissection was performed on 27 patients, and 6 patients underwent radioiodine (RAI) therapy. Results of surgical pathology showed that 95 CLNMs of 375 lymph nodes were diagnosed in 27 patients.

### 3.2. Valuable Indicators of Basic Characteristics, Conventional US, CDFI, and CEUS

Basic characteristics of the PTC patients are shown in Table 1. Metastasis history on first thyroidectomy was significantly different between the patients with and without CLNM (*P* < 0.05). The characteristics such as mean age, gender, multiple nodules, and mean FNA period after operation did not show significant differences between the two groups (all *P* > 0.05).

With regard to conventional US and CEUS features, intensity at peak time, homogeneity, CDFI patterns, echogenicity, and cystic change or microcalcification were significantly associated with CLNM, whereas shape, fatty hilum, position, and entrance mode in CEUS were not (Table 2).

### 3.3. Multivariate Logistic Regression Analysis

Multivariate logistic regression analysis showed that homogeneity (OR = 53.9, *P* ≤ 0.001), intensity at peak time (OR = 26.4, *P* ≤ 0.001), and cystic change or calcification (OR = 11.9, *P* > 0.05) were the three strongest independent predictors for CLNM (Table 3).

### 3.4. ROC Analysis

The overall diagnostic performance of homogeneity (Az: 0.845; 95% CIs: 0.767–0.923) was superior to other predictors such as intensity at peak time (Az: 0.733; 95% CIs: 0.634–0.832), CDFI patterns (Az: 0.689; 95% CIs: 0.582–0.797), and cystic change or calcification (Az: 0.645; 95% CIs: 0.568–0.722) (Table 4).

A multivariate logistic regression equation was established with the following significant predictive factors: *P* = 1/1 + exp∑[−3.213 + 2.77 *∗* cystic or calcification + 0.13 *∗* CDFI patterns +3.65 *∗* homogeneity + 2.43 *∗* intensity at peak time]. The diagnostic value of the equation was discriminative with an area under the ROC curve of 0.927 (95% CI: 0.874–0.980). Ultimately, the sensitivity and specificity were 77.8% and 100.0%, respectively.

## 4. Discussion

**Figure 1:**
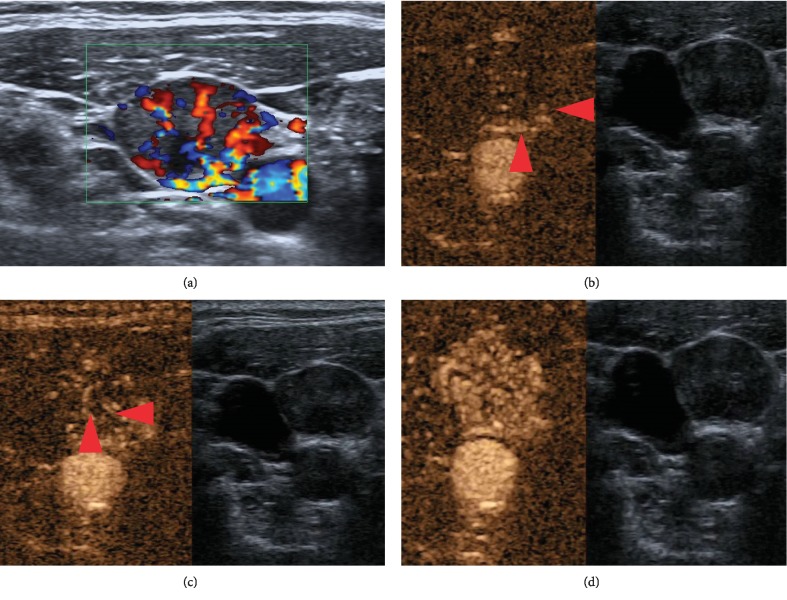
Fifty-one-year-old PTC woman had received left thyroidectomy two years ago. ACLNs were detected on the right neck, located in level IV. (a) Loss of fatty hilum, hyperechogenicity, and oval in conventional US (17 × 10 mm). A peripheral (Type IV) vascular pattern was shown in CDFI. Compared with previously conventional sonographic figures, aberrant vessels were clearly shown in chronological order in CEUS figures. (b) Microbubbles rose at the nodal capsule (red arrows) in the beginning (11 s after SonoVue injection). (c) Then aberrant vessels developed from the nodal capsule to hilum (red arrows, 13 s after SonoVue injection). (d) LN showed hyperenhancement 18 s after SonoVue injection. And this ACLN was proved to be metastatic PTC by FNA.

Although controversy still exists that CLNM has no major impact on PTC specific survival after thyroidectomy, surgical excision of locoregional disease was recommended in combination with RAI therapy for patients with stable or slowly progressive asymptomatic disease. Conventional US is recommended as a primary screening tool for PTC patients, while FNA is further recommended for ACLN in guidelines [3, 10]. Sonographic feature overlaps limit the use of conventional US: round shape, loss of fatty hilum, or hyperechogenicity could exist in both benign nodules and CLNMs (Figure 1). The presence of calcifications and cystic change shows too low specificity, so we had to merge them as one in the present study (Table 2).

**Figure 2:**
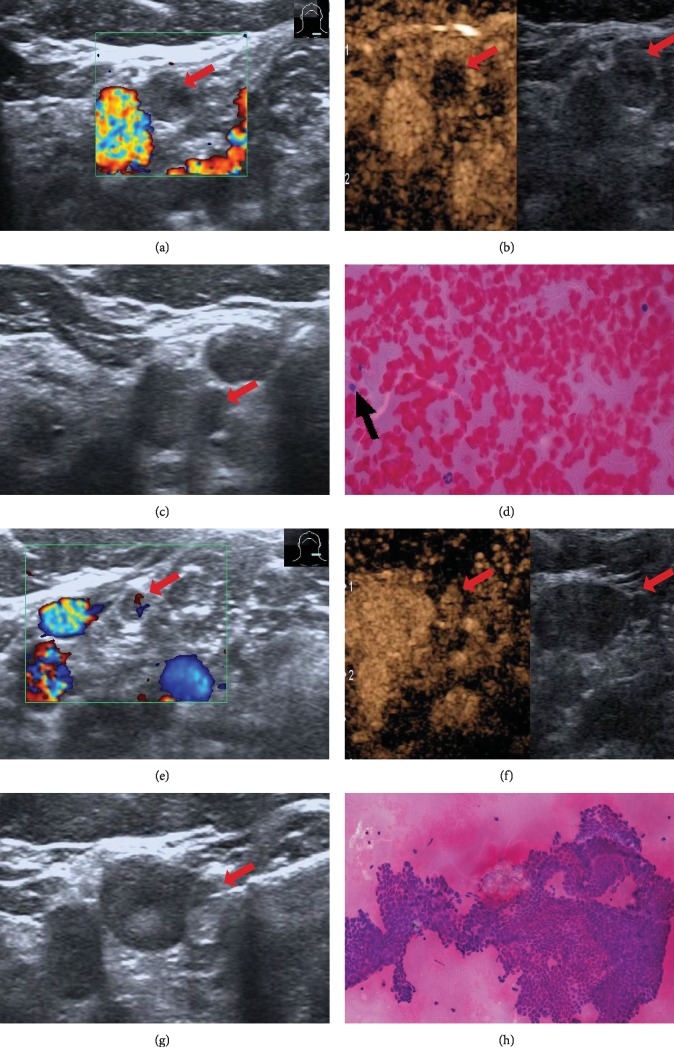
Twenty-eight-year-old PTC woman had received left thyroidectomy two years ago. ACLNs were detected on the left neck twice by conventional US in consecutive follow-up. (a) ACLN located in level IV: loss of fatty hilum and round shape (6 × 5 mm) in conventional US. None CDFI was shown (Type I). (b) Hypoenhancement on CEUS (13 s after SonoVue injection). (c) Needle was inserted in ACLN. (d) FNA revealed scattered normal lymph cell in a blood cell background. (e) Another ACLN located in level II: loss of fatty hilum and round shape (4 × 4 mm) in conventional US. A normal hilar (type II) pattern is shown in CDFI. (f) Hyperenhancement on CEUS (15 s after SonoVue injection). (g) Needle was inserted in ACLN. (h) FNA diagnosis was metastatic PTC.

Color Doppler flow pattern is a good tool to distinguish benign and malignant nodes. Histopathological studies indicate that arteries and veins enter the node at the hilum and spread in benign nodes. By comparison, most CLNMs have aberrant vessels with a curved course entering from the nodal capsule to hilum (Figure 1). Theoretically, on CDFI, benign nodes tend to manifest hilar vascularity or appear avascular, while CLNMs tend to have peripheral or mixed vascularity [11, 12]. However, because CDFI suffers from technical limitations with regard to visualizing fine vessels and low-velocity blood flow, in many situations, CDFI was not sensitive to internal flow of LNs, as the LNs were tiny (Figure 2).

CEUS has the capability to clearly display microvascular blood flow in tumors and can accurately evaluate the sequence and intensity of tumor perfusion and vascularity [13]. Many relevant issues of CEUS in PTC-related neck LN metastasis had been published due to the development of more advanced ultrasonographic equipment and the introduction of second-generation contrast agents (SonoVue). Studies are focused on the CEUS features of PTC themselves to predict the neck LN metastasis. Zhang et al. [14] revealed that the heterogeneous hypoechoic enhancement in PTC was the most valuable in predicting CLNM. On the contrary, Liu et al. found that partial hyperenhancement in early ascending CEUS period was significantly higher in LNM + group than that in LNM-group [15]. As angiogenesis plays an important role in the development, growth, and metastases of PTC, it was hypothesized in our previous study [16] that the higher enhancements display at peak time is, the greater the microvascular density in PTC and the greater the possibility of neck LN metastasis may happen. Recently, CEUS has been used for the assessment of lymph nodes in PTC patients preoperatively. Hong [17] revealed that CLNM more often manifested centripetal perfusion, hyperenhancement, heterogeneous enhancement, perfusion defects, and ring-enhancing margins than benign lymph nodes while Chen et al. [18] considered that centripetal perfusion was the most meaningful feature predicting CLNM. Similarly, homogeneity and intensity were also the two strongest independent predictors for CLNM postoperatively in the present study. The recurrence diagnosis by CEUS determines the further management of PTC patients and even causes a second surgical treatment.

**Figure 3:**
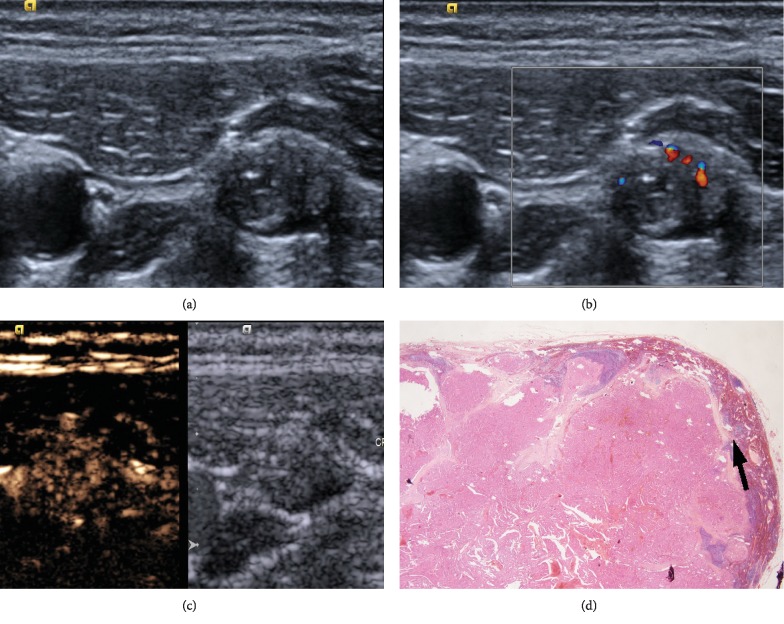
Forty-one-year-old man had received left thyroidectomy four years ago. ACLNs were detected on the left neck one year ago. (a) ACLN located in level IV: loss of fatty hilum, microcalcification, and hyperechogenicity in conventional US (15 × 7 mm). A normal hilar (Type II) pattern is shown. Notice: right lower edge of LN showed hypoechogenicity. (b) Heterogeneous hyperenhancement on CEUS (19 s after SonoVue injection). Notice: right lower edge of LN showed hypoenhancement.) (c) Needle was inserted in ACLN, and FNA diagnosis was metastatic PTC. (d) This CLNM was filled with cancerous thyroid tissue under optical microscopy, and normal lymph tissues still exist in the edge of LN.

Different from previous literature which pointed that lymph node metastasis showed hyperenhancement [19–21], CLNM of PTC showed that heterogeneous enhancement and hyperenhancement in CEUS depend on its pathological characteristics: the CLNMs were filled with cancerous tissues with the characteristics of the thyroid follicular epithelium under optical microscopy. And the thyroid follicular epithelium is a rich blood supplied tissue showing hyperenhancement on CEUS; moreover, normal lymph cells still exist in CLNM, and normal lymph tissue had weaker peak intensity than cancerous tissue which cause heterogeneous pattern (Figure 3). As for entrance mode, most of lymph nodes were too tiny to recognize the entrance mode of CEUS (benign group median size: 10 × 5 mm; CLNM group median size: 12 × 6 mm).

**Figure 4:**
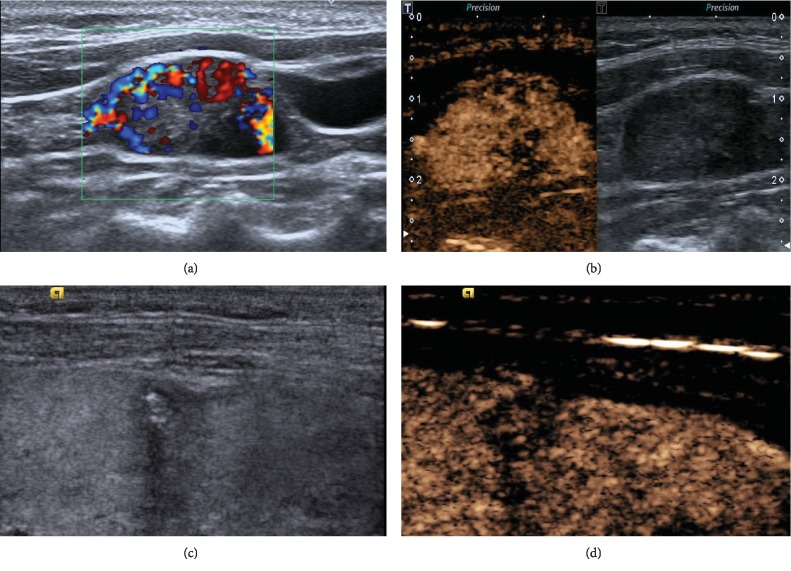
Fifty-five-year-old woman had undergone right thyroidectomy three years ago. ACLNs were detected on the left neck one year ago. (a) ACLN located in level I: loss of fatty hilum and hyperechogenicity in conventional US (23 × 13 mm). A peripheral (Type IV) vascular pattern with evidence of abundant atypical peripheral flow is shown in CDFI. (b) Heterogeneous hyperenhancement on CEUS (13 s after SonoVue injection). (c) A hypoechoic, solid, and calcified thyroid nodule in the right lobe (TI-RADS category 4b) three years ago before the first thyroidectomy. (d) It showed hyperenhancement in CEUS (18 s after SonoVue injection) on stored video.

Several clinical phenomena had recently aroused the interest of examiners. Firstly, in most CLNM group patients (24/33) was found metastasis on the first thyroidectomy which implied that preoperative CLNM patients have greater possibility of having second metastasis even if they received RAI therapy after operation. Secondly, we reviewed primary PTC lesions on stored video and found that most PTCs preoperatively showed hypoenhancement on CEUS (Figure 4). Thirdly, ACLNs detected by conventional US or CEUS only cover a small proportion of lymph nodes existing (86/375).

Previous studies [14–18] focused on the use of CEUS to evaluate the presence of LNs in humans with PTC, and safety assessments were not the main point of these studies. Safety of CEUS by intravascular administration has been studied extensively [22–24]: the patients with the sentinel LN of breast cancer received their subdermal implants, and the region around the injection sites was massaged to accelerate the uptake of contrast agent into the lymphatic system which may cause a metastatic spreading risk. In the present study, the safety profile is excellent without incidence of adverse experiences—another factor may be the low-dosage of contrast agent (1.2 ml). Our study is not without limitations. First, not all malignant lymph nodes were confirmed by neck dissection—six patients only choose radioiodine therapy. Second, the sample size of the present study is relatively small since ACLN was defined as nodes showing at least two suspicious US features instead of one feature in previous literature [4, 8]. Third, CEUS also had limitation based on our data. Seven benign LNs were misdiagnosed by CEUS (showing heterogeneous enhancement, hyperenhancement) in this study.

## 5. Conclusions

In a word, conventional US is the primary tool for cervical evaluation of PTC patients postoperatively. CEUS will be further recommended once ACLNs are found. Heterogeneous enhancement and hyperenhancement are useful criteria to distinguish malignant LNs from benign ones.

## Figures and Tables

**Table 1:** Basic characteristics of 56 PTC patients with ACLN after thyroidectomy.

Characteristics	Total PTCs with ACLN (*n* = 56)		*P*
	CLNM group (*n* = 33)	Benign group (*n* = 23)	
Mean age	50.2 ± 2.7	56.1 ± 1.9	0.111
Range of age	20–79	30–73	
Gender			0.385
Male	11 (19.6%)	5 (8.9%)	
Female	22 (39.3%)	18 (32.2%)	
Multiple LNs			0.282
Yes	24 (42.9%)	16 (28.5%)	
No	9 (16.1%)	7 (12.5%)	
Mean FNAB period after operation (month)	56.3 ± 16.3	37.0 ± 7.4	0.352
Metastasis in history			0.007
Yes	24 (42.9%)	8 (14.3%)	
No	9 (16.1%)	15 (26.7%)	

**Table 2:** Conventional US, CDFI, and CEUS indicators of ACLN (*n* = 86).

US feature	Benign	CLNM	*P* value
	(*n* = 32)	(*n* = 54)	
Median size	10 × 5 mm	12 × 6 mm	1.000
Shape			0.171
Round	16 (18.6%)	36 (41.9%)	
Oval	16 (18.6%)	18 (20.9%)	
Fatty hilum			0.101
Absent	18 (20.9%)	40 (46.5%)	
Present	14 (16.3%)	14 (16.3%)	
Echogenicity			0.067
Hyper	8 (9.3%)	25 (29.1%)	
Hypo	24 (27.9%)	29 (33.7%)	
Cystic change or microcalcification			0.004
Present	2 (2.3%)	19 (22.1%)	
Absent	30 (34.9%)	35 (40.7%)	
Position			0.366
Level II	3 (3.5%)	7 (8.1%)	
Level III	8 (9.3%)	11 (12.8%)	
Level IV	19 (22.1%)	29 (33.7%)	
Level V	1 (1.2%)	0 (0%)	
Level VI	1 (1.2%)	7 (8.1%)	
CDFI patterns			0.014
Type I	20 (23.3%)	16 (18.6%)	
Type II	3 (3.5%)	7 (8.1%)	
Type III	3 (3.5%)	4 (4.7%)	
Type IV	6 (7.0%)	27 (31.4%)	
Intensity at peak time			≤0.001
Hyperenhancement	10 (11.6%)	42 (48.8%)	
Hypoenhancement	22 (25.6%)	12 (14.0%)	
Entrance mode			0.142
Centripetal	6 (7.0%)	19 (22.1%)	
Noncentripetal	26 (30.2%)	35 (40.7%)	
Homogeneity			≤0.001
Heterogeneous	4 (4.6%)	44 (51.2%)	
Homogeneous	28 (32.6%)	10 (11.6%)	

**Table 3:** Multivariate logistic regression analysis in predicting CLNM.

Overall (*n* = 86)	B	SE	*P*	Odds ratio	95% CI
Cystic change or calcification	2.78	1.17	0.018	16.1	1.62–158.8
CDFI patterns	0.13	0.29	0.648	1.14	0.64–2.06
Intensity at peak time	2.43	0.93	0.009	11.4	1.82–71.2
Homogeneity	3.65	0.85	0.000	38.6	7.25–204.9

**Table 4:** ROC analyses for the characteristics in prediction of CLNM from patients with PTCs.

Overall (*n* = 86)	Az	95% CI	Sen (%)	Spe (%)	PPV (%)	NPV (%)	Accuracy (%)
Cystic change or calcification	0.645	0.568–0.722	35.2	93.8	90.5	46.2	57.0
CDFI patterns	0.689	0.582–0.797	70.4	62.5	76.0	55.6	67.4
Intensity at peak time	0.733	0.634–0.832	77.8	68.8	80.8	64.7	74.4
Homogeneity	0.845	0.767–0.923	81.5	87.5	91.6	73.7	83.7
US	0.746	0.647–0.848	83.3	56.2	74.1	56.2	67.4
CEUS	0.895	0.838–0.952	64.8	100.0	100	62.7	77.9
Predictive equation	0.936	0.885–0.986	87.0	87.5	92.2	80.0	87.2

## Data Availability

All data generated or analyzed during this study are included in this article.
